# Outcomes of Joint-Preserving Surgery for Rheumatoid Forefoot Deformity: An Editorial

**DOI:** 10.3390/ijerph19042038

**Published:** 2022-02-11

**Authors:** Koichiro Yano, Katsunori Ikari

**Affiliations:** 1 Institute of Rheumatology, Tokyo Women’s Medical University Hospital, Tokyo 162-0054, Japan; 2 Department of Orthopedic Surgery, Tokyo Women’s Medical University, Tokyo 162-0054, Japan; 3 Division of Multidisciplinary Management of Rheumatic Diseases, Tokyo Women’s Medical University, Tokyo 162-0054, Japan

In the past few decades, physicians have been able to effectively manage patients with rheumatoid arthritis (RA) thanks to advances in treatment strategies including molecular-targeting drugs. By controlling the disease activities, we have also been able to suppress the progression of joint destruction, and maintain patients’ physical functions. The surgical procedures available to patients with RA have also advanced. Although most surgeries for RA patients have been performed for the destruction of major joints, the number of surgeries for minor joints, especially for the feet, has recently increased. This is because the aim of surgeries has changed from increasing the activities of daily living to improving functions and correcting deformities. This Special Issue focuses on joint-preserving surgeries for forefoot deformities in patients with RA. Joint-preserving surgery corrects forefoot deformities while preserving the metatarsophalangeal (MTP) joint. This procedure is different from traditional joint-sacrificing surgeries, such as resection arthroplasty, silicon implant replacement, and joint arthrodesis.

Our research team published our first literature review on joint-preserving surgeries in 2021 [1], but the first article on joint-preserving surgeries for RA-affected feet was published in 1999 by Hanyu et al. [2]. Most articles relating to joint-preserving surgeries were published in the 2010s. Some studies have compared joint-preserving and joint-sacrificing surgeries, showing that joint-preserving surgery was equivalent or superior to joint-sacrificing surgery.

Lee et al. [3] compared plantar pressure, the visual analog scale for pain (pain VAS) and general health (general VAS), hallux valgus angle (HVA), the angle comprising the first metatarsal and the second metatarsal (M1M2A), and the angle comprising the first metatarsal and the fifth metatarsal (M1M5A), preoperatively and postoperatively after joint-preserving surgery involving the modified Mitchell’s osteotomy of the first metatarsal and shortening oblique osteotomy of the four lateral metatarsals. They showed that pain VAS, general VAS, HVA, M1M2A, and M1M5A scores improved significantly after surgery. In the analysis of preoperative and postoperative plantar pressure, the peak pressure increased significantly at the first metatarsophalangeal (MTP) joint and decreased significantly at the second and third MTP joints. The outcomes of the plantar pressure analysis in this study were comparable to those of previous studies. Considering the clinical and radiographic outcomes in this study, joint-preserving surgery can be considered highly effective for forefoot deformities in patients with RA.

Etani et al. [4] described the outcomes of joint-preserving surgery involving the modified scarf osteotomy for the great toe, and metatarsal shortening offset osteotomy for the lesser toes in patients with RA. The follow-up period was relatively long, at 4.6 years (2-7 years). All subscales of the Japanese Society for Surgery of the Foot (JSSF) standard rating system and a self-administered foot evaluation questionnaire (SAFE-Q) improved significantly after joint-preserving surgery. In particular, JSSF scores showed a remarkable improvement after surgery, and JSSF hallux and lesser toe scores improved from 41.1 and 29.3 preoperatively to 88.4 and 85.2 postoperatively, respectively. Radiographic angle measurements of the forefoot also significantly improved. The rate of the hallux valgus (HV) recurrence was 7.5%. It should be noted that no cases of recurrent HV deformity required revision surgery. The authors also found that preoperative disease activity scores showed a significant negative correlation with postoperative JSSF scores (P=0.04), using logistic regression analysis. This indicates that higher disease activity is associated with worse clinical outcomes. Moreover, the spread of preoperative M2M5A was a risk factor for postoperative resubluxation of the lesser toe. The spread of M2M5A indicates excessive loading on the lateral part of the foot, leading to postoperative resubluxation of the lesser toes. This study highlighted the importance for surgeons to comprehensively understand and recognize foot deformities.

Matsumoto et al. [5] assessed the outcomes of scarf and Akin osteotomy with intra-articular stepwise lateral soft tissue release in patients with RA. Intra-articular stepwise lateral soft tissue release involves three steps: (1) dissection of the lateral metatarsosesamoid suspensory ligament; (2) lateral capsulotomy at the joint level; (3) tenotomy of the adductor tendon insertion into the proximal phalanx. The stepwise lateral release was performed until the M1M2A spontaneously corrected the HV deformity. After release, osteotomy of the first metatarsal was performed. All subscales of the JSSF and SAFE-Q improved significantly after joint-preserving surgery, with a mean follow-up period of 32.0 months. The authors suggested that sequential lateral soft tissue release, beginning with the dissection of the lateral metatarsosesamoid suspensory ligament, was effective and did not require a complete release of the abductor hallucis tendon or the transverse metatarsal ligament. There was only one case with severe hallux varus after surgery. It was one of three cases in which all three steps of lateral soft tissue release were performed. The authors concluded that the stepwise intra-articular approach enabled minimum lateral soft tissue release and thereby contributed to the low prevalence of severe iatrogenic hallux varus that required revision procedures.

Takakubo et al. [6] compared joint-preserving surgery with joint-sacrificial surgery in patients with RA. In this study, joint-sacrificing surgery was performed if severe subluxation and dislocation of the MTP joint with contractures and stiffness were observed on the manipulation or stress radiographs. The mean follow-up was 9.4 years. The JSSF score of the joint-preserving surgery group was significantly higher than that of the joint-sacrificing surgery group at the latest follow-up. However, the authors mentioned the possibility of selection bias because the surgical procedure was performed at the surgeon’s discretion. The HV recurrence rate in each group was relatively high. The authors discussed that one of the reasons for this was the difference in indications for joint-preserving surgery. Their cohort included patients with challenging cases involving high disease activity and severe joint destruction, particularly those who underwent joint-preserving surgery before disease management using biologics.

Ohashi et al. [7] suggested that there is no standardized radiographic measurement method to evaluate the MTP joint deformities or outcomes of these surgeries. They established a practical grading method for MTP joint deformities based on MTP overlap distance (MOD). MOD was defined as the distance between the tip of the metatarsal head and the most proximal point of the base of the proximal phalanx along the metatarsal axis. Receiver operating characteristic (ROC) analysis of MOD predicting toes of the foot without any symptoms (pain or callosities) and toes with callosities indicated that the ROC area under the curve (AUC) was 0.89, the 95% CI was 0.86–0.92, and the optimal cut-off value was 7.8 mm. Additionally, the authors described the validity of MOD as a tool for evaluating the outcomes of joint-preserving surgery. Unfortunately, the MOD cutoff value for remnant plantar callosities was not assessed in this study because of the limited number of study samples. We expect that further utility of MOD as an MTP assessment tool before and after surgery will be researched in the future. 

Surgeries for forefoot deformities in patients with RA have changed dramatically from joint-sacrificing surgery to joint-preserving surgery. In Japan, orthopedic surgeons assume the role of rheumatologists, and have the responsibility of managing patients with RA. Considering a paradigm shift in RA medications, the surgeons also focused surgeries toward joint preservation. While all the articles in this Special Issue came from Japan, we strongly hope that this Special Issue can serve as a guide for joint-preserving surgeries in patients with RA for orthopedic surgeons worldwide.

## Data Availability

All data is available through cited publications.
